# Screening and Validation of Significant Genes with Poor Prognosis in Pathologic Stage-I Lung Adenocarcinoma

**DOI:** 10.1155/2022/3794021

**Published:** 2022-04-11

**Authors:** Yujie Deng, Xiaohui Chen, Chuanzhong Huang, Jun Song, Sisi Feng, Xuzheng Chen, Ruixiang Zhou

**Affiliations:** ^1^School of Basic Medical Sciences, Fujian Medical University, Fuzhou 350108, Fujian, China; ^2^Key Laboratory of Gastrointestinal Cancer, Ministry of Education, Fujian Medical University, Fuzhou 350108, Fujian, China; ^3^Molecular Oncology Research Institute, The First Affiliated Hospital of Fujian Medical University, Fuzhou 350005, Fujian, China; ^4^Department of Thoracic Surgery, Fujian Medical University Cancer Hospital, Fujian Cancer Hospital, Fuzhou, Fujian Province 350014, China; ^5^College of Clinical Medicine for Oncology, Fujian Medical University, Fuzhou, China; ^6^Laboratory of Immuno-Oncology, Fujian Medical University Cancer Hospital, Fujian Cancer Hospital, Fuzhou, Fujian 350014, China

## Abstract

**Background:**

Although more pathologic stage-I lung adenocarcinoma (LUAD) was diagnosed recently, some relapsed or distantly metastasized shortly after radical resection. The study aimed to identify biomarkers predicting prognosis in the pathologic stage-I LUAD and improve the understanding of the mechanisms involved in tumorigenesis.

**Methods:**

We obtained the expression profiling data for non-small cell lung cancer (NSCLC) patients from the NCBI-GEO database. Differentially expressed genes (DEGs) between early-stage NSCLC and normal lung tissue were determined. After function enrichment analyses on DEGs, the protein-protein interaction (PPI) network was built and analyzed with the Search Tool for the Retrieval of Interacting Genes (STRING) and Cytoscape. Overall survival (OS) and mRNA levels of genes were performed with Kaplan–Meier analysis and Gene Expression Profiling Interactive Analysis (GEPIA). qPCR and western blot analysis of hub genes in stage-I LUAD patients validated the significant genes with poor prognosis.

**Results:**

A total of 172 DEGs were identified, which were mainly enriched in terms related to management of extracellular matrix (ECM), receptor signaling pathway, cell adhesion, activity of endopeptidase, and receptor. The PPI network identified 11 upregulated hub genes that were significantly associated with OS in NSCLC and highly expressed in NSCLC tissues compared with normal tissues by GEPIA. Elevated expression of ANLN, EXO1, KIAA0101, RRM2, TOP2A, and UBE2T were identified as potential risk factors in pathologic stage-I LUAD. Except for ANLN and KIAA0101, the hub genes mRNA levels were higher in tumors compared with adjacent non-cancerous samples in the qPCR analysis. The hub genes protein levels were also overexpressed in tumors. In vitro experiments showed that knockdown of UBE2T in LUAD cell lines could inhibit cell proliferation and cycle progression.

**Conclusions:**

The DEGs can probably be used as potential predictors for stage-I LUAD worse prognosis and UBE2T may be a potential tumor promoter and target for treatment.

## 1. Introduction

Lung cancer is still the leading cause of cancer incidence and mortality both in China and worldwide [1, 2]. Although the 5-year survival rate of stage-I non-small cell lung cancer (NSCLC) is between 70% and 92%, there is still much progress such as screening, early detection, and genome analyses that have been made for lung cancer [3, 4], and they shed light on the possibility of developing more reliable prognostic biomarkers and sensitive predisposing genes in the carcinogenesis of lung cancer, better understanding the underlying mechanism and improving the treatment effect. Although more and more pathologic stage-I non-small cell lung cancer (NSCLC) patients had been diagnosed and cured, some of them still suffered from early relapse and distant metastasis after surgery. Thus, the discrimination of specific biomarkers to predict the clinical outcome of early-stage NSCLC patients is indispensably necessary. Many researchers had worked out a variety of schemes in the prediction of resectable lung cancer patients [5, 6], while few focused on the outcome foretelling especially in pathologic stage-I patients. The use of gene chips can quickly detect differentially expressed genes (DEGs) within cancerous and normal tissues, identifying novel genetic predictors of lung cancer, facilitating improvements to early detection, and elucidating the mechanisms influencing carcinogenesis [7].

Ubiquitin-conjugating enzyme E2T (UBE2T) is a member of the E2 family in the ubiquitin-proteasome pathway that is located on chromosome 1q32.1. As one of the post-translational modifications, the ubiquitin-proteasome system regulates protein ubiquitination and stability and is recognized as a key regulator of cell proliferation, invasion, and differentiation [8]. UBE2T plays an important role in the Fanconi anemia pathway [9] by ubiquitinating FANCD2 and inducing the DNA damage response (DDR). Overexpression of UBE2T has been detected in different tumor types. UBE2T promotes tumor progression by downregulation of BRCA1 in breast cancer [10] and p53 ubiquitination in hepatocellular carcinoma cells [11]. However, the role of UBE2T in early-stage LUAD remains unclear. In vitro, we found that UBE2T promoted the proliferation of LUAD cells, which verified its functions.

## 2. Materials and Methods

### 2.1. Patients and Tissue Samples

The study was approved by the Ethics Committee of Clinical Research of Fujian Cancer Hospital. LUAD and paired non-cancerous tissues were obtained from seven patients diagnosed with stage-I lung adenocarcinoma who underwent surgical resection at Fujian Cancer Hospital between March 2014 and December 2014. All the patients were pathologically confirmed. None of the patients had received prior radiotherapy or chemotherapy. Fresh frozen samples were stored at −80°C.

### 2.2. Microarray Data Acquisition and DEGs Data Processing

Gene expression profile of GSE18842, GSE31210, and GSE33532 NSCLC and normal lung tissues were obtained from NCBI-GEO. All these microarray data were derived from GPL570 platforms ((HG-U133_Plus_2) Affymetrix Human Genome U133 Plus 2.0 Array). GSE18842 included 46 tumors and 45 controls; GSE31210 included 226 lung adenocarcinomas and 20 normal lung tissues; and GSE33532 had 80 tumors and 20 matched normal lung tissues. DEGs were identified via GEO2R online tools. The DEGs between NSCLC and normal lung tissue were selected by the criteria of │logFC│ > 2 as well as an adjusted *P* value <0.05. The raw data in TXT format were analyzed in Venn software online (http://bioinformatics.psb.ugent.be/webtools/Venn/) to evaluate the commonly DEGs within these 3 data sets. The DEGs with logFC > 0 was taken as upregulated genes and logFC < 0 as downregulated genes.

### 2.3. Gene Oncology and PPI Network Analysis

Gene ontology analysis (GO) is used to define genes and their RNA or protein products to identify unique biological properties. The Database for Annotation, Visualization, and Integrated Discovery (David) was utilized to determine these DEGs enrichment, including molecular function (MF), cellular component (CC), biological process (BP), and Kyoto Encyclopedia of Gene and Genome (KEGG) pathways (*P* < 0.05). Protein-protein interaction (PPI) was constructed via STRING (https://www.string-db.org/). The STRING database was used to determine the potential correlation between these DEGs. Then Cytoscape (version 3.7.1) was applied to visualize the PPI network. Modules of the PPI network was validated by the MCODE app in Cytoscape (degree cutoff = 2, node score cutoff = 0.2, *k*-core = 2, and max. depth = 100).

### 2.4. RNA Expression of Core Genes and Survival Analysis

Gene Expression Profiling Interactive Analysis (GEPIA) website was applied to analyze the DEGs mRNA expression between NSCLC and normal tissues (*P* < 0.05). Kaplan–Meier plotter (http://kmplot.com/analysis/index.php?p=service&cancer=lung) was used to determine the effect of genes on survival based on GEO (Affymetrix microarrays only). Survival within groups was compared by log-rank estimates (*P* < 0.05).

### 2.5. ROC Curve, Forest Plot, and Volcano Plot

The receiver operating characteristic curve (ROC) analysis was applied to evaluate the specificity and sensitivity of the core genes. The pROC R packages were installed, and the area under the curve (AUC) and *P* value were calculated (TCGA). Forest plot R packages were installed and the forest plot of subgroup analysis related to the stage of the candidate genes was drawn. The ggplot2 R packages were installed to draw the volcano plot labeled with hub genes.

### 2.6. Human Protein Atlas

The Human Protein Atlas (https://www.proteinatlas.org/) is an online website that includes pathology atlas of nearly 20 types of malignant tumors. In our study, immunohistochemical data of ANLN, TOP2A, and RRM2 were used to compare the expression in normal and lung adenocarcinoma tissues. The intensity of antibody staining indicated the protein expression of hub genes.

### 2.7. Real-Time Quantitative Reverse Transcription Polymerase Chain Reaction and Western Blot Analysis

Total RNA of fresh frozen tissues and cells were isolated using TRIzol reagent (Invitrogen) and was transferred to cDNA using Evo M-MLV RT Kit with gDNA Clean for qPCR (Accurate biology, AG). The SYBR® Green Premix Pro Taq HS qPCR Kit (Accurate biology, AG) and ROX Reference Dye (4 *μ*M) (Accurate biology, AG) were used to perform PCR amplification on Agilent Mx3000p real-time PCR system. The primers were synthesized by Sunya Biotechnology Co. Ltd. (Fuzhou, China); GAPDH was used as the internal control. Each measurement was performed in triplicate. The expression levels of hub gene mRNAs were evaluated using a relative quantification approach (2^−ΔΔCt^ method) against GAPDH levels. Many more details of primer sequences for qRT-PCR were in a supplementary appendix online (Supplementary Table S2).

The cell lines were collected and lysed on ice with radioimmunoprecipitation assay (RIPA) buffer containing 0.1 mg/ml PMSF (Sangon Biotech, Shanghai) and cocktail (MCE). The fresh frozen tissues from seven patients paired with lung adenocarcinoma (T) and adjacent non-cancerous control tissues (N) were minced into small pieces before being lysed. The protein lysates were obtained from the supernatant through centrifugation at 12,000 g for 20 min at 4°C. The total amount of protein for each sample was 25 *μ*g, run on 8%–12% gradient SDS‐polyacrylamide gels, and then transferred onto a PVDF membrane (Immobilon-P^SQ^, Millipore, Merck, USA). The membranes were probed with primary antibodies at 4°C overnight after blocking with 0.5% BSA blocking buffer for 1 h at room temperature. The membranes were then incubated with the appropriate secondary antibodies at room temperature for 1 hour and finally were detected by using an ECL blotting analysis system (ImageQuant LAS 4000 mini, GE, USA). The details of antibodies information can be found in Supplementary Table S3).

### 2.8. In Vitro Experiment

#### 2.8.1. Cell Culture and siRNA Transfection

The A549 and H1299 LUAD cell lines obtained from Laboratory of Radiation Oncology and Radiobiology, Fujian Medical University Cancer Hospital, were cultured in RPMI-1640 (cytiva) containing 10% fetal bovine serum (Biological Industries) with 100 units/mL penicillin and 100 *μ*g/mL streptomycin (Gibco) in a humidified 5% CO_2_ incubator at 37°C. Three small interfering RNAs (si-UBE2T) against UBE2T (si-UBE2T-homo-192, 5′-CUCCUCAG AUCCGAUUUCUTT-3′; si-UBE2T-homo-374, 5′-GCUGACAUAUCCUCAGAA UTT-3′; and si-UBE2T-homo-97, 5′-CCUGCGAGCUCAAAUAUUATT-3′) and negative control siRNAs (si-NC, 5′-UUCUCCGAACGUGUCACGUTT), which were obtained from GenePharma (Shanghai, China), were transfected into cell lines using siRNA-mate transfection reagent (GenePharma, Shanghai, China) according to the manufacturer's instructions.

#### 2.8.2. Cell Proliferation Assays

Cell proliferation was assessed by Cell Counting Kit-8 (CCK-8) and colony‐forming assays. A total of 5 × 10^3^ transfected cells in 100 *μ*L medium per well were added to a 96‐well plate for 4, 8, 24, 48, and 72 hours. At the indicated times, 10 *μ*L (at a concentration of 10%) CCK-8 solution (Vazyme, Nanjing, China) was added to each well and incubated for 1 hour at room temperature. The absorbance was assessed at a 450 nm wavelength under a plate reader (BioTek ELx800). For the Colony‐forming assays, transfected A549 and H1299 cell lines were seeded (1 × 10^3^ cells/well) into six-well plates with 2 mL complete medium and divided into an si-NC and si-UBE2T groups. The colonies were fixed with 4% methanol (Solarbio) after 7–10 days of culture and then stained with 0.1% crystal violet solution (Biosharp, China). After 15 min, the cells were washed gently with PBS 3 times and then air‐dried. Finally, the colony‐forming units (consisting of ≥50 cells) were observed under an inverted microscope (ZEISS Primo Vert) and counted using ImageJ software. All experiments were performed in triplicate.

#### 2.8.3. Flow Cytometry

LUAD cells were added into 6‐well plates at a density of 1.3 × 10^5^ cells per well for transfection after 24 hours incubation. Transfected cells were digested by 0.25% trypsin-EDTA (Gibco), collected into a centrifuge tube, and then fixed in 70% precooled ethanol overnight at −20°C. The cells were washed twice with PBS. After recollection by centrifugation at 1,500 rpm for 5 min, the cells were stained by 500 *μ*L PI/RNase staining buffer (BD, USA). Culturing for 15 min at 37°C in dark, the cells were analyzed by LSRFortessaX-20 (BD Biosciences). All experiments were performed in triplicate.

### 2.9. Statistical Analysis

SPSS 18.0, GraphPad Prism 8.0, R software (version 4.0.2), and ModFit LT were used to conduct the analysis and generate graphs. The in vitro experiments were repeated in triplicate, and all data from the experiments were expressed as mean ± SE. *T*-test was used to evaluate the statistical significance of differences between experimental groups. A paired-samples *t*-test was used to assess the difference in hub genes expression between LUAD and non-cancerous tissues. ^*∗*^*P* < 0.05 was considered statistically significant.

## 3. Results

### 3.1. Identification of DEGs in NSCLCs

**Figure 1:**
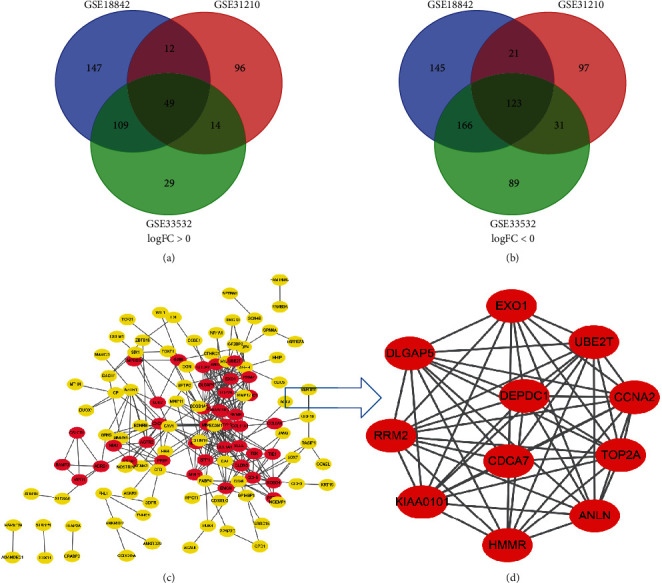
A total of 172 DEGs in the data sets (GSE18842/GSE31210/GSE33532) via the Venn diagrams website and PPI network constructed by STRING online platform and Cytoscape software. (a and b) 49 and 123 DEGs were upregulated (logFC > 0) and downregulated (logFC < 0) in the three data sets, respectively. (c) A total of 119 DEGs in the PPI network complex. Nodes: proteins; edges: interaction of proteins; red nodes were upregulated DEGs; and and yellow ones were downregulated DEGs. (d) Module analysis via Cytoscape software (degree cutoff = 2, node score cutoff = 0.2, *k*-core = 2, and max. depth = 100).

**Figure 2:**
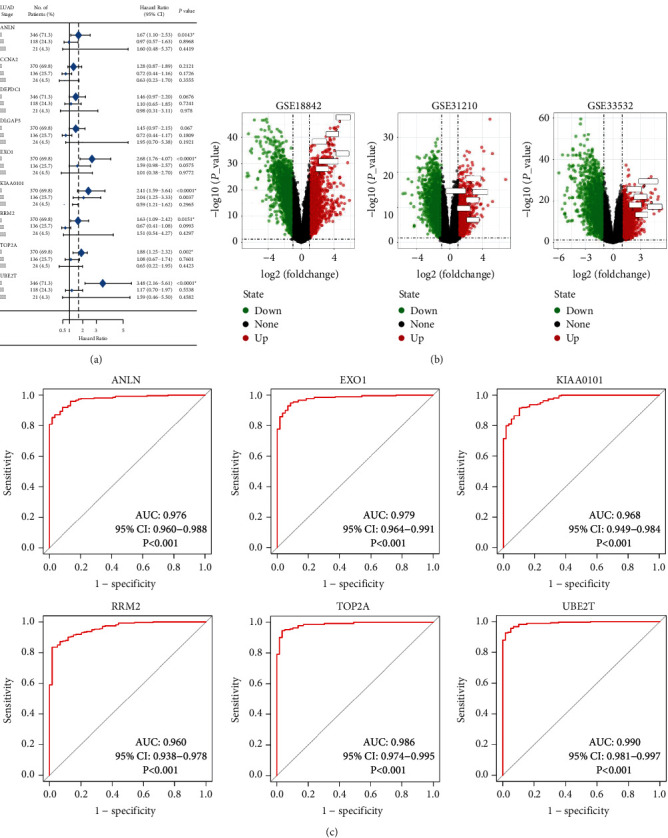
The OS for stage-I–III LUAD of nine candidate genes and ROC analysis: (a) six genes had a significantly worse survival in stage-I lung adenocarcinoma, while three had no significant (^*∗*^*P* < 0.05); (b) the distribution of all DEGs and six genes in volcano plots including GSE18842, GSE31210, and GSE33532; and (c) the ROC curves of six genes in pathologic stage-I LUAD. ROC: receiver operating characteristic and AUC: area under the curve.

In total, 352 NSCLC and 85 normal lung tissues were included. A total of 1,044, 626, and 818 DEGs were extracted from GSE18842, GSE31210, and GSE33532 by GEO2R online tool, respectively (Table S1 and Table 1). A total of DEGs including 49 upregulated genes (logFC > 0) and 123 downregulated genes (logFC < 0) were determined through Venn diagram software (Figures 1(a)–1(b) and 2(b)).

### 3.2. DEGs Gene Ontology Analysis in NSCLCs

All 172 DEGs analyzed by David online tools and GO analysis indicated roles in biological process (BP), cell component (CC), and molecular function (MF). For BP, upregulated DEGs were enriched in the regulation of collagen catabolic process, extracellular matrix disassembly, proteolysis, collagen fibril organization, sensory perception of sound, and inner ear morphogenesis, and downregulated DEGs were mainly enriched in angiogenesis, vasculogenesis, cell surface. For CC, proteinaceous ECM, collagen trimer, extracellular region, and space were the main function that the upregulated DEGs were enriched in. As indicated in Table 2, downregulated DEGs consisted mainly of integral component of plasma membrane, membrane raft, and integral component of membrane. And molecular function of the DEGs majorly lay in metalloendopeptidase activity, endopeptidase activity, serine-type endopeptidase activity, and receptor activity.

### 3.3. PPI Network and Modular Analysis

All 172 DEGs were imported into the network that screened a total of 119 nodes and 283 edges, including 39 upregulated and 80 downregulated genes (Figure 1(c)). Fifty-three out of 172 DEGs were not in the DEGs PPI network. Then Cytoscape MCODE analysis demonstrated 11 core nodes among the 119, which were all upregulated genes (Figure 1(d)).

### 3.4. Analysis of Core Genes by GEPIA and the Kaplan–Meier Plotter

**Figure 3:**
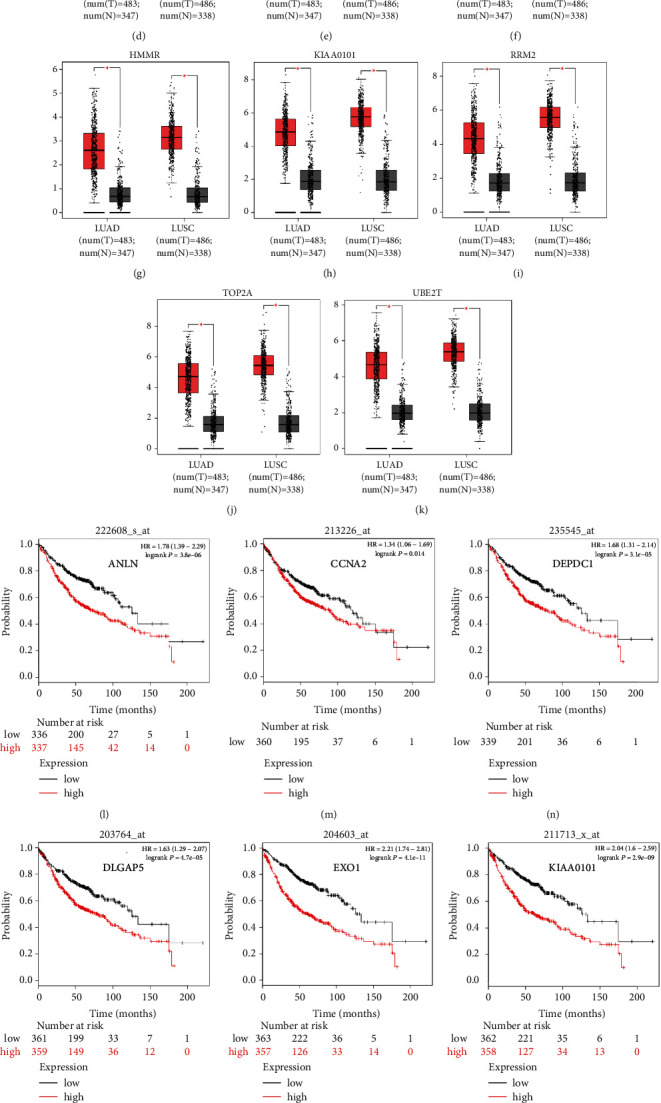
The expression of the 11 hub genes analyzed by the GEPIA website and the prognosis identified by Kaplan–Meier plotter online tools. (a–k) All the 11 genes demonstrated enhanced expression in both LUAD and LUSC compared to the normal specimen (^*∗*^*P* < 0.05). Red and grey color stood for tumor and normal lung tissue, respectively. (l–t) Nine of 11 genes had a significantly worse survival (*P* < 0.05) in LUAD.

The expression level of the 11 core genes among cancerous as well as normal lung tissues was assessed via GEPIA, showing that in comparison to normal lung tissue, ANLN, CCNA2, CDCA7, DEPDC1, DLGAP5, EXO1, HMMR, KIAA0101, RRM2, TOP2A, and UBE2T were indeed highly expressed in both adenocarcinoma and squamous cell cancerous tissue (Figures 3(a)–3(k)). Kaplan–Meier plotter was used to identify the prognostic values of these 11 core genes, demonstrating all 11 genes were significantly correlated with worse prognosis and shorter OS (Table 3) in NSCLC patients. These 11 genes were then individually studied the different roles that would play in the different histology of NSCLC, finding that none of them demonstrated a significant effect on OS in lung squamous cell carcinoma (LUSC; Table 3), while the other 9 genes, including ANLN, CCNA2, DEPDC1, DLGAP5, EXO1, KIAA0101, RRM2, TOP2A, and UBE2T, demonstrated potential in the prediction of survival based on the expression level in LUAD (Figures 3(l)–3(t)) rather than CDCA7 and HMMR (Table 3).

Further analysis was then managed to uncover the prognostic effect of these genes on different pathologic stages of lung adenocarcinoma patients. Interestingly, the results of forest plot showed that genes such as ANLN (HR = 1.67; 95% CI: 1.1–2.53; *P*=0.0143), EXO1 (HR = 2.68; 95% CI: 1.76–4.07; *P* < 0.0001), KIAA0101 (HR = 2.41; 95% CI: 1.59–3.64; *P* < 0.0001), RRM2 (HR = 1.63; 95% CI: 1.09–2.42; *P*=0.0151), TOP2A (HR = 1.88; 95% CI: 1.25–2.32; *P*=0.002), and UBE2T (HR = 3.48; 95% CI: 2.16–5.61; *P* < 0.0001) demonstrated significantly prognostic effect in early disease, especially in pathologic stage-I lung adenocarcinoma patients. The risk ratio (HR) for UBE2T is the most obvious. In addition, KIAA0101 also exhibited potential in the prediction of OS in stage-II LUAD patients (HR = 2.04; 95% CI: 1.25–3.33; *P*=0.0037). CCNA2, DEPDC1, and DLGAP5 demonstrated no difference in different pathologic stage patients (Figure 2(a)). The distributions of six hub genes have been labeled in volcano plot (Figure 2(b)).

### 3.5. ROC Curves of the Candidate Genes

According to ROC curve analysis, in the pathologic stage-I LUAD, the AUCs of ANLN, EXO1, KIAA0101, RRM2, TOP2A, and UBE2T were 0.976 (95% CI: 0.960–0.988), 0.979 (95% CI: 0.964–0.991), 0.968 (95% CI: 0.949–0.984), 0.960 (95% CI: 0.938–0.978), 0.986 (95% CI: 0.974–0.995), and 0.990 (95% CI: 0.981–0.997), respectively (Figure 2(c); *P* < 0.001).

### 3.6. The 6 Hub Genes Were up-Regulated in Stage-I LUAD Compared with Normal Lung Tissues

**Figure 4:**
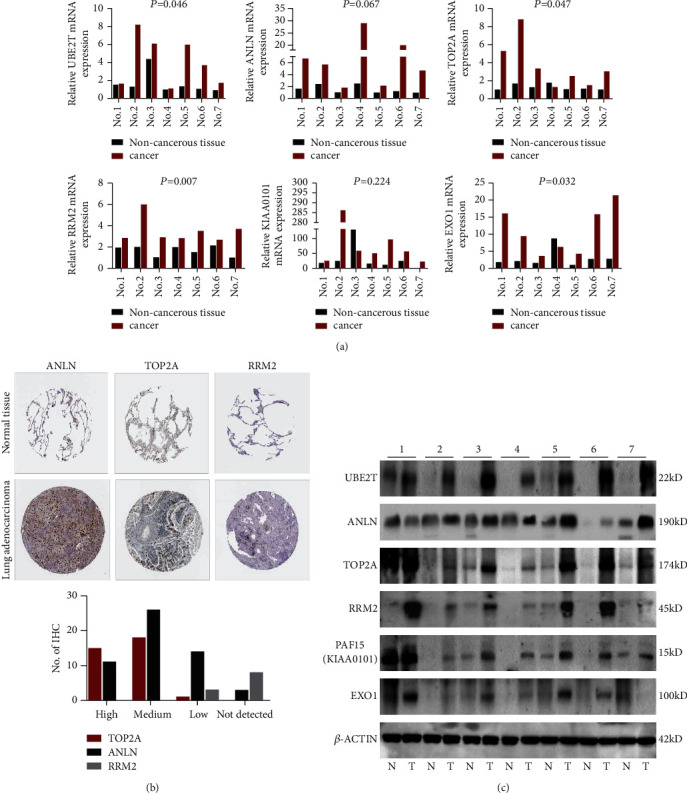
The expression of six genes in stage-I LUAD: (a) the mRNA level expressions of six genes were analyzed in lung adenocarcinoma and adjacent non-cancerous control samples from seven patients, using qRT‐PCR (^*∗*^*P* < 0.05); (b) immunohistochemical analysis of ANLN, TOP2A, and RRM2 in normal and lung adenocarcinoma tissues from the Human Protein Atlas (HPA); and (c) western blot of six markers protein level expression in stage-I lung adenocarcinoma (T) and adjacent non-cancerous control samples (*N*) from seven patients. *β*-ACTIN was used as an internal control.

To further determine the clinical significance of the six hub genes, we investigated the expression of UBE2T, ANLN, TOP2A, RRM2, KIAA0101, and EXO1 in seven randomly selected pairs of stage-I LUAD and adjacent non-cancerous tissues. The seven patients' characteristics were listed in Table 4. qRT‐PCR analysis showed that mRNA expression of UBE2T (*P*=0.046), TOP2A (*P*=0.047), RRM2 (*P*=0.007), and EXO1 (*P*=0.032) were significantly higher in the LUAD tissues than in the adjacent non-cancerous tissues (Figure 4(a)). We tried to explore the protein expression of hub genes using Human Protein Atlas (HPA) after studying the mRNA expression. Immunohistochemistry assays from HPA showed that TOP2A and RRM2 protein was not expressed in normal lung tissues. TOP2A staining was high or medium in most LUAD tissues compared to low. However, RRM2 was not detected in eight LUAD tissues. There were also some IHC data of ANLN showing high and medium staining in cancer samples, although mRNA expression levels were not significantly different between cancerous and non-cancerous samples in our seven patients (Figure 4(b)). Finally, we investigated the protein expression of UBE2T, ANLN, TOP2A, RRM2, KIAA0101 (PAF15), and EXO1 in the seven pairs of tissues. The results from western blot analysis also indicated that these six hub genes were overexpressed in tumor samples (Figure 4(c)). These results indicated that hub genes are overexpressed in stage-I LUAD and might promote tumor genesis.

### 3.7. Validation of UBE2T in Vitro That Promoted LUAD Cell Proliferation

Interestingly, the hazard ratio (HR) of UBE2T was the most obvious. And both mRNA and protein levels showed differences between LUAD and adjacent non-cancerous tissues.

**Figure 5:**
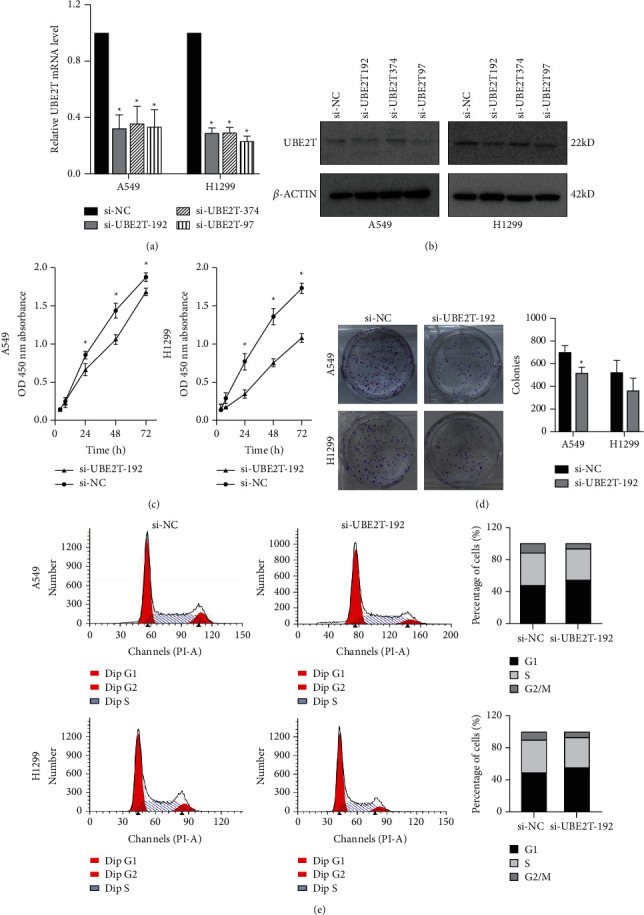
UBE2T promotes the malignant biological behaviour of LUAD cells: (a and b) the relative mRNA and protein expression in A549 and H1299 cell lines after being transfected with small interfering RNAs (siRNAs) against UBE2T, by qRT-PCR (^*∗*^*P* < 0.05); (c) CCK-8 assay showed the inhibition of proliferation ability of LUAD cells with transient UBE2T knockdown (^*∗*^*P* < 0.05); (d) clone formation assay showed the inhibition of proliferation ability of LUAD cells with transient UBE2T knockdown (^*∗*^*P* < 0.05); and (e) flow cytometry showed G0/G1 arrested in LUAD cells with transient UBE2T knockdown. si‐UBE2T group: A549 and H1299 cell lines transfected with si-UBE2T vector and si‐NC group: LUAD cells transfected with control vector.

To explore the biological function of UBE2T in LUAD progression, A549 and H1299 cells with transient UBE2T knockdown were established. We transfected LUAD cells with three independent small interfering RNAs (siRNAs) and a negative control vector (si‐NC group; Table 5). Transfection efficiency was verified in UBE2T knockdown cells using real-time quantitative PCR and western blot (Figures 5(a) and 5(b)). Compared with the si‐NC group, the mRNA expression level of UBE2T in A549 cells was markedly reduced by the transfection of the si-UBE2T vectors (Figure 5(a)). UBE2T protein expression was effectively downregulated in si-UBE2T-192 transfected A549 cell line than that of si-UBE2T-374 and si-UBE2T-97 (Figure 5(b)). The same results were observed in H1299 cell lines (Figures 5(a) and 5(b)). So we chose si-UBE2T-192 for further experiments in vitro.

The results from the Cell Counting Kit-8 (CCK-8) assays revealed that UBE2T knockdown (si-UBE2T-192) significantly reduced the LUAD cell lines' proliferative ability (Figure 5(c)). Similarly, the colony-forming assays suggested that UBE2T knockdown inhibited A549 cell line clonogenic ability. We also detected the tendency of decreased number of colonies after being transfected with si-UBE2T-192 in an H1299 cell line, although there was no statistically significant difference (Figure 5(d)). In addition, we analyzed cell cycle distribution using flow cytometry and showed that decreased UBE2T inhibited cell cycle progression with the accumulation of LUAD cell lines in G1-phase and reduction in S-phase (Figure 5(e)). These results demonstrated that as one of the 6 hub genes, UBE2T depletion did inhibit the proliferation of LUAD cells in vitro, and it might be a potential biomarker for early-stage LUAD diagnosis and prognosis.

## 4. Discussion

With the development of lung cancer screening and low-dose CT (LDCT) scan technology, many pathologic stage-I non-small cell lung cancer (NSCLC) patients have been diagnosed and cured. However, some still suffered from early relapse and distant metastasis after surgery, and few researchers focused on the outcome foretelling especially in pathologic stage-I LUAD patients. Therefore, there is a substantial need for novel therapeutic targets. In this study, bioinformatics analysis was performed to identify the candidate core genes correlated with early-stage LUAD.

We analyzed RNA sequencing data from the three profile data sets of early-stage NSCLC from the GEO data sets (GSE18842, GSE31210, and GSE33532) via GEO2R and Venn software, discriminated 172 DEGs including 49 upregulated and 123 downregulated genes compared to normal lung tissue. The 172 DEGs were detected by GO terms analyses. The BP of upregulated DEGs was particularly enriched in the management of extracellular matrix (ECM) that facilitated tumor metastasis, and that of downregulated DEGs was mainly enriched in cell surface receptor signaling pathway, cell adhesion, and receptor internalization, which might in part accelerate cellular detachment and eventually promote distant metastasis. The CCs of upregulated DEGs were also enriched in proteinaceous ECM, extracellular region, and space. For MF, DEGs were significantly focused on the activity of endopeptidase and receptor. The GO terms analysis revealed that the DEGs were obviously associated with ECM-related functions. A previous study showed that the extracellular matrix has crucial roles in lung cancer metastasis [12, 13]. Next, the DEGs PPI network of 119 nodes and 283 edges was built, and eventually, 11 out of 39 upregulated genes were screened. Further validation of these genes via GEPIA analysis indicated that all 11 genes exhibited higher expression levels in both histologies (LUAD and LUSC) of NSCLC in comparison to normal lung tissue. We then evaluated their prognostic effect on NSCLC patients via Kaplan–Meier plotter analysis and found them having a significantly worse survival. Interestingly, final analyses showed that none of the 11 genes had any significance on the outcome of patients with LUSC histology (all *P* > 0.05), while 6 of the 11 genes (ANLN, EXO1, KIAA0101, RRM2, TOP2A, and UBE2T) demonstrated statistical significance on worse prognosis in patients with pathologic stage-I LUAD histology (all *P* < 0.05). Chen et al. [14] also verified that UBE2T and KIAA0101 were highly expressed in early-stage lung adenocarcinoma through bioinformatic analysis and experiments in vitro. Moreover, to explore the predictive ability of the six hub genes, the ROC curves were performed. Notably, all six genes enabled a relatively high capacity for discrimination stage-I LUAD patients, with better clinical accuracy and higher diagnostic value.

Much effort has been tried to discriminate different genetic subgroups of surgically resected pathologic stage-I NSCLCs that would probably relapse and metastasize, including gene panel biomarkers [3, 15] and tumor genotyping [16, 17]. In the present study, we demonstrated that enhanced expression of either ANLN, EXO1, KIAA0101, RRM2, TOP2A, or UBE2T genes in pathologic stage-I LUAD patients was a risk factor of inferior outcome and shorter OS, although this finding might need further validation in larger sample size or in real-world studies.

ANLN overexpression correlated with worse outcomes in a wide spectrum of malignancies including lung [18–21], breast [22], and gastric cancer [23]. ANLN expression [18] was essential for the growth of lung cancer cell lines, as well as the maintenance of cellular motility and cytokinesis. Interestingly, the endogenous ANLN could be detected in various patterns of localization, either in nuclei and/or cytoplasm, and NSCLC patients with nuclear localization of ANLN had a significantly worse outcome compared to the cytoplasmic pattern. Intracellular ANLN level was found to change dynamically during mitosis, increase at a transition period from G1 to S phase, peak at S phase, and decrease in G2/M phase. The reduction of ANLN induced apoptosis and thus inhibited tumor proliferation in pancreatic cancer [24]. ANLN downregulation inhibited cell migration and invasion in breast cancer, which was considered a biomarker for global genomic instability and to play a vital role in replicative immortality of tumor cells. Based on its adverse prognostic effect onstage-I LUAD patients, we speculated that ANLN over-expression might probably be an early event in the carcinogenesis of NSCLC.

Exonuclease 1 (EXO1) gene locates at 1q42–43 and encodes an 846 amino acid protein [25]. Owing to its role in DNA repair, maintenance of chromatin stability, and modulation of DNA recombination, the relationship between polymorphisms of EXO1 and the risk of cancer had been well studied, with at least nine genetic variants identified [26–29]. However, its expressions in carcinogenesis and prognosis in cancer entities were limited. Several studies indicated EXO1 was remarkably overexpressed and correlated with unfavorable patient prognosis in the colorectum, liver, pancreas, prostate, and so on [30–33]. However, the expression and prognostic value of EXO1 in NSCLC especially early-stage LUAD remains undefined, although some reported several EXO1 SNPs were correlated with worse prognosis in patients with NSCLC [27]. Here, we defined enhanced EXO1 expression as a risk factor in pathologic stage-I LUAD patients.

High expression of KIAA0101 (proliferating cell nuclear antigen (PCNA) associated factor 15 (PAF15)), containing a PCNA-binding motif and playing a key role in DNA repair, cellular apoptosis, and cell cycle, had been observed in a variety of human tumors including lung cancer [34–37]. High KIAA0101 level was significantly associated with shorter survival in NSCLC patients, especially in LUAD [34], which was consistent with our findings that KIAA0101 was bioinformatically identified as a negative prognostic factor in patients with pTNM stage-I (HR: 2.41; 95% CI: 1.59–3.64; *P* < 0.0001) and stage-II (HR: 2.04; 95% CI: 1.25–3.33; *P*=0.0037). As a potential cell proliferation-related factor, KIAA0101 might probably become a treatment target either in human nasopharyngeal carcinoma [38] or in lung cancer patients with poor response to immune checkpoint inhibitors (ICIs) [35]. Further validation of this finding in real-world prospective studies would be necessary for our future studies.

Ribonucleotide reductase M2 subunit (RRM2), a small subunit of the ribonucleotide reductase complex that acts as an oncogenic role under pathological conditions, and its overexpression was found in various cancers including NSCLCs [39, 40]. Tabbal et al. [41] revealed that RRM2 overexpression was associated with poor prognosis and inhibition of RRM2 blocked cell proliferation, induced apoptosis, and inhibited cell migration. Recent studies also rendered RRM2 as a target in anti-cancer drug designation for treatment with anti-RRM2 drugs could reduce ribonucleotide reductase activity and consequently decreased the synthesis of dNTPs with concomitant inhibition of DNA replication, arrest of cells at S-phase, DNA damage, and finally apoptosis [42].

Topoisomerase-II alpha (TOP2A) is an essential nuclear enzyme regulating the topological state of DNA during transcription and is involved in the processes of chromosome condensation and chromatid separation [43]. As a marker of proliferation and chemotherapy resistance, a higher TOP2A level was indicative of poor prognosis in many human cancers and also the target for some most widely used anti-cancer drugs [44, 45]. A recent study [46] found that resistance of esophageal cancer cells to paclitaxel can be reduced by the knockdown of the long non-coding RNA DDX11-AS1 through TAF1/TOP2A inhibition. Wang et al. [47] revealed that TOP2A had prognostic significance in early-stage lung cancer patients, and its expression correlated with the levels of immune cell infiltration, especially dendritic cells.

UBE2T (ubiquitin-conjugating enzyme, E2T), a typical ubiquitin-conjugating enzyme, connects with a particular E3 ubiquitin ligase to degrade related substrates [48]. In normal lung tissue, basal cells of pseudostratified ciliated columnar epithelium with high self-renewal and differentiation ability showed positive UBE2T immunohistochemistry staining, suggesting that UBE2T was closely related to cell proliferation [49]. UBE2T not only involved in DNA repair [50] but also regulated the protease in the glucose metabolism of tumor tissue, leading to its ubiquitination and degradation, ultimately promoting the tumor by glucose metabolism [51, 52]. UBE2T knockdown inhibited NSCLC proliferation and invasion by suppressing the Wnt/b-catenin signaling pathway [53]. Tu et al. [20] found that high UBE2T and ANLN expression correlated with worse outcomes in NSCLCs, regardless of their histology. Neither their histologic features nor combined diseases had been clarified, which was quite different from the results of our study.

We verified both the mRNA and protein expression levels of the six hub genes in stage-I LUAD. It was determined that EXO1, RRM2, TOP2A, and UBE2T expression was significantly upregulated in stage-I LUAD patients. Although there was no significant difference in the mRNA expression of ANLN and KIAA0101 between tumor and normal lung tissues, the tendency of increased relative mRNA expression could be detected, which also were probably ascribed to much few of the matching specimens. Interestingly, the hazard ratio (HR) of UBE2T was the most obvious. To further support the results of our bioinformatics analyses, we carried out UBE2T-related in vitro experiments. The proliferative ability and cell cycle progression of LUAD cell lines were inhibited after the knockdown of the UBE2T in A549 and H1299 cell lines. These results indicated the hub genes might be potential biomarkers for early-stage LUAD diagnosis and prognosis and played a vital role in stage-I LUAD. UBE2T overexpression might also promote cancer development. Nevertheless, more stage-I tumor samples would be needed to verify the expression of the hub genes. We also planned to verify the gene function in vitro and in vivo in our further study, and the underlying molecular mechanisms of the hub genes in the development and progression of early-stage LUAD remain to be further explored.

## 5. Conclusion

Our bioinformatic analyses identified six DEGs (ANLN, EXO1, KIAA0101, RRM2, TOP2A, and UBE2T) that could probably be used as potential biomarkers in the prediction of worse clinical outcomes in surgically resected stage-I LUADs and could facilitate the selection of some defined patients with a higher risk of postoperative relapse or distant metastasis. We also concluded that UBE2T enhanced LUAD cells' proliferative ability and cell cycle progression. The finding claims further validation with a larger sample size and underlying molecular mechanisms of the hub genes in the development and progression of early-stage LUAD.

## Figures and Tables

**Table 1:** Patients' demographic characteristics of three GEO data sets.

GEO accession	GSE18842	GSE31210	GSE33532
No. of patients			
Normal	45	20	20
Tumor	46	226	80
Mean age (years)	NR	59	64
Gender			
Male	NR	105	64
Female		121	16
Histology			
LUAD	14	226	40
LUSC	32	0	16
Others	0	0	24
pTNM stage			
I	38	168	56
II	4	58	24
III-IV	4	0	0
Smoking history			
Yes	NR	122	NR
No		104	
Driven gene status			
EGFR mutation	NR	127	NR
KRAS mutation		20	
EML4-ALK fusion		11	
Triple negative		68	

**Table 2:** Gene ontology analysis of DEGs in NSCLC.

Expression	Category	Term	Count	*P*-value	FDR
Upregulated	GOTERM_BP_DIRECT	GO:0030574∼collagen catabolic process	8	4.6*E* − 1 0	6.5*E* − 7
	GOTERM_BP_DIRECT	GO:0022617∼extracellular matrix disassembly	6	2.2 *E* − 6	0.003069
	GOTERM_BP_DIRECT	GO:0007605∼sensory perception of sound	6	3.4*E* − 5	0.047394
	GOTERM_BP_DIRECT	GO:0030199∼collagen fibril organization	4	1.7*E* − 4	0.244070
	GOTERM_BP_DIRECT	GO:0006508∼proteolysis	7	0.002578	3.540562
	GOTERM_BP_DIRECT	GO:0042472∼inner ear morphogenesis	3	0.009302	12.233335
	GOTERM_CC_DIRECT	GO:0005578∼proteinaceous extracellular matrix	8	5.2*E* − 06	0.005507
	GOTERM_CC_DIRECT	GO:0005581∼collagen trimer	5	9.2*E* − 05	0.096644
	GOTERM_CC_DIRECT	GO:0005576∼extracellular region	13	6.1*E* − 04	0.638880
	GOTERM_CC_DIRECT	GO:0005615∼extracellular space	9	0.020704	19.761271
	GOTERM_MF_DIRECT	GO:0004222∼metalloendopeptidase activity	6	1.08*E* − 05	0.012389
	GOTERM_MF_DIRECT	GO:0004175∼endopeptidase activity	4	3.7*E* − 04	0.425928
	GOTERM_MF_DIRECT	GO:0004252∼serine-type endopeptidase activity	6	5.1*E* − 04	0.578533
	GOTERM_MF_DIRECT	GO:0003682∼chromatin binding	6	0.003359	3.771756

Downregulated	GOTERM_BP_DIRECT	GO:0001525∼angiogenesis	11	7.1*E* − 07	0.001095
	GOTERM_BP_DIRECT	GO:0001570∼vasculogenesis	5	3.2*E* − 04	0.492091
	GOTERM_BP_DIRECT	GO:0007166∼cell surface receptor signaling pathway	8	0.001115	1.718397
	GOTERM_BP_DIRECT	GO:0007155∼cell adhesion	10	0.001443	2.218739
	GOTERM_BP_DIRECT	GO:0031623∼receptor internalization	4	0.002008	3.075026
	GOTERM_BP_DIRECT	GO:0002576∼platelet degranulation	5	0.003101	4.711874
	GOTERM_CC_DIRECT	GO:0005887∼integral component of plasma membrane	24	7.3*E* − 06	0.008643
	GOTERM_CC_DIRECT	GO:0045121∼membrane raft	8	2.3*E* − 04	0.267090
	GOTERM_CC_DIRECT	GO:0016021∼integral component of membrane	49	2.5*E* − 04	0.292185
	GOTERM_CC_DIRECT	GO:0005886∼plasma membrane	41	4.8*E* − 04	0.562068
	GOTERM_CC_DIRECT	GO:0009897∼external side of plasma membrane	7	0.001690	1.978880
	GOTERM_CC_DIRECT	GO:0016324∼apical plasma membrane	8	0.001739	2.035373
	GOTERM_MF_DIRECT	GO:0004872∼receptor activity	6	0.007296	9.063121
	GOTERM_MF_DIRECT	GO:0008201∼heparin binding	5	0.012256	14.785944
	GOTERM_MF_DIRECT	GO:0044325∼ion channel binding	4	0.025214	28.204372

**Table 3:** The expression and prognosis of 11 core genes.

Category	Genes
Highly expressed genes in NSCLCs compared to normal tissues (*P* < 0.05)	ANLN CCNA2 CDCA7 DEPDC1 DLGAP5 EXO1 HMMR KIAA0101 RRM2 TOP2A UBE2T
Genes with significantly worse OS in NSCLC (*P* < 0.05)	ANLN CCNA2 CDCA7 DEPDC1 DLGAP5 EXO1 HMMR KIAA0101 RRM2 TOP2A UBE2T
Genes without significantly worse OS in LUSC (*P* < 0.05)	ANLN CCNA2 CDCA7 DEPDC1 DLGAP5 EXO1 HMMR KIAA0101 RRM2 TOP2A UBE2T
Genes with significantly worse OS in LUAD (*P* < 0.05)	ANLN CCNA2 DEPDC1 DLGAP5 EXO1 KIAA0101 RRM2 TOP2A UBE2T

OS, overall survival; LUSC, lung squamous cell carcinoma; and LUAD, lung adenocarcinoma.

**Table 4:** The seven patients' characteristics of the fresh frozen samples.

# No.	Age (y. o.)	Sex	Smoking history/brinkman index	Location	Surgery	TNM	Histology/predominant growth patterns	TTF-1	Bronchial involvement/lymphovascular invasion	Relapse	Status
No. 1	52	F	No	LLL	VATS radical resection	T2aN0M0, IB	Ade/acinar + micropapillary	(+)	No	No	Alive
No. 2	58	M	Yes/400	LUL	Left pneumonectomy	T1aN0M0, IA1	Ade/acinar	(+)	No	No	Alive
No. 3	59	F	No	LLL	VATS radical resection	T1cN0M0, IA3	Ade/acinar	NA	No	No	Alive
No. 4	48	F	No	RUL	VATS radical resection	T2aN0M0, IB	Ade/acinar + papillary	(+)	No	Yes (bone/liver/brain)	Dead
No. 5	48	M	Yes/300	LLL	VATS radical resection	T1cN0M0, IA3	Ade/acinar + micropapillary + solid	(+)	No	No	Alive
No. 6	61	M	Yes/200	LLL	VATS exploration plus radical resection	T1bN0M0, IA2	Ade/acinar + solid	(+)	No	Yes (local)	Alive
No. 7	64	F	No	RUL	VATS radical resection	T2aN0M0, IB	Ade/acinar	NA	No	No	Alive

y. o., years old; F, female; M, male; LLL, left lower lobe; LUL, left upper lobe; RUL, right upper lobe; VATS, video-assisted thoracoscopic surgery; TTF-1, positive thyroid transcription factor-1; and NA, no application.

**Table 5:** Sequence of si-UBE2T and si-negative control group.

Category	Forward primer (5′------3′)	Reverse primer (5′------3′)
si-UBE2T-192	CUCCUCAGAUCCGAUUUCUTT	AGAAAUCGGAUCUGAGGAGTT
si-UBE2T-374	GCUGACAUAUCCUCAGAAUTT	AUUCUGAGGAUAUGUCAGCTT
si-UBE2T-97	CCUGCGAGCUCAAAUAUUATT	UAAUAUUUGAGCUCGCAGGTT
si-NC	UUCUCCGAACGUGUCACGUTT	ACGUGACACGUUCGGAGAATT

## Data Availability

The data sets generated and/or analyzed during the current study are available in the Gene Expression Omnibus: GEO accession viewer (nih.gov); STRING: functional protein association networks (string-db.org); Gene Expression Profiling Interactive Analysis: GEPIA (cancer-pku.cn); Kaplan–Meier plotter (Lung; kmplot.com); and The Human Protein Atlas. All data can be obtained from the first author or corresponding author.

## Abbreviations

LUAD: Lung adenocarcinoma
DEGs: Differentially expressed genes
STRING: Search tool for the retrieval of interacting genes
PPI: Protein-protein interaction
GEPIA: Gene expression profiling interactive analysis
ROC: Receiver operating characteristic curve.

## Supplementary Materials

Supplementary Materials Supplementary Table 1: A total of 172 differentially expressed genes (DEGs) were detected from the three data sets, including 49 upregulated and 123 downregulated genes in NSCLC tissues compared to normal tissues. Supplementary Table 2: Sequence of primers for quantitative reverse transcription-polymerase chain reaction. Supplementary Table 3: Antibodies for western blot analysis.
