# Supplementary material for: Screening and Validation of Significant Genes with Poor Prognosis in Pathologic Stage-I Lung Adenocarcinoma
Source: J Oncol. 2022 Apr 11;2022:3794021. doi: 10.1155/2022/3794021 (PMC9015852; doi:10.1155/2022/3794021)
Supplement: Supplementary Materials — Supplementary Table 1: A total of 172 differentially expressed genes (DEGs) were detected from the three data sets, including 49 upregulated and 123 downregulated genes in NSCLC tissues compared to normal tissues. Supplementary Table 2: Sequence of primers for quantitative reverse transcription-polymerase chain reaction. Supplementary Table 3: Antibodies for western blot analysis. [file 3794021.f1.docx]

Table S1 172 differentially expressed genes(DEGs) were detected from the three datasets, including 49 up-regulated and 123 down-regulated genes in NSCLC tissues compared to normal tissues.

| DEGs | Genes Name |
| --- | --- |
| Up-regulated | *CDH3 IGF2BP3 IGSF9 HMGB3 MMP3 FERMT1 CRABP2* ***HMMR*** *PROM2 CXCL13 ANKRD22 AKR1B10* ***EXO1*** *TMPRSS4 HS6ST2 SPP1 COL1A1 NMU ADAMDEC1* ***ANLN*** *GJB6 SIX1 COL10A1 LRRC15 GPR87* ***CDCA7*** *COL11A1 PLPP2 CTHRC1* ***RRM2 TOP2A*** ***KIAA0101*** *GJB2 GREM1 SPRR1A* ***CCNA2*** *CP CST1 MMP1 MMP12* ***UBE2T*** *KRT15 TFAP2A* ***DLGAP5*** *MMP9* ***DEPDC1*** *MMP11 FAM83A ARNTL2* |
| Down-regulated | *HBA2///HBA1 RTKN2 EMCN SOX7 GPIHBP1 MFAP4 KCNT2 PEBP4 SLC6A4 PECAM1 KCNK3 MMRN2 NOSTRIN NCKAP5 OGN SCARA5 CLDN5 BTNL9 IGSF10 SCGB1A1 CDO1 HIGD1B CA4 SDPR TEK GRK5 ID4 EXOSC7///CLEC3B DACH1 LOC100653057///CES1 FAM150B ACKR1 STXBP6 NR4A1 LYVE1 ADAMTS8 GDF10 LEPROT///LEPR AKAP12 CD36 FAM162B GPD1 HSPA12B ROBO4 SPTBN1 CALCRL CAV1 RASIP1 PPBP JAM2 PTPRB FOXF1 ACADL ANKRD29 PIR-FIGF///FIGF AQP4 NEBL MT1M TNNC1 MCEMP1 HBB SERTM1 SELE FHL1 CPB2 SSTR1 FAM189A2 SORBS2 LRRN3 ABCA8 AOC3 CCM2L SFTPC ADRB1 TCF21 TGFBR3 HHIP ADH1B ARHGEF26 ZBTB16 ASPA FABP4 EDNRB SCN4B FCN3 ZBED2 MYCT1 KANK3 STX11 LINC00312 FAM107A CCDC85A PLAC9 CCBE1 AGER MARCO CD300LG TIE1 MMRN1 AGTR1 VIPR1 WIF1 RAMP3 CLIC5 FGFR4 FHL5 MAMDC2 CAMK2N1 AGTR2 CLDN18 C2orf40 CDH5 PDK4 GPM6A COL6A6 CFD GKN2 LRRC36 CYP4B1 HYAL1 TMEM100 DUOX1 AFF3* |

Table S2 Sequence of primers for quantitative reverse transcription polymerase chain reaction.

| Gene | Forward primer (5’------3’) | Reverse primer(5’------3’) |
| --- | --- | --- |
| UBE2T | ATTGATTCTGCTGGAAGGAT | TGAGGAAGGCTGGCTTAT |
| ANLN | TCTTAACGGTGATGCTCTG | GCCTTGGACTTGGATGTT |
| TOP2A | ATGGTGGCAAGGATTCTG | ACGCTGGTTGTCATCATAT |
| RRM2 | GAACTTATTAGCAGAGATGAGG | TTCTATCCGAACAGCATTGA |
| KIAA0101 | TTCCACCTCTGCCACTAAT | ACTGCTTCCTGCCTCTTC |
| EXO1 | TTCCAGACAAGGCAACAG | AGACCTCCAGACCAACTAA |
| GAPDH | TATGACAACAGCCTCAAGAT | AGTCCTTCCACGATACCA |

Table S3 Antibodies for western blot analysis.

| Antibody | Concentration | Specificity | Company |
| --- | --- | --- | --- |
| UBE2T | 1:1000 | Rabbit monoclonal | Abcam140611 |
| ANLN | 1:1000 | Mouse monoclonal | Santa Cruz Biotechnology sc-271814 |
| TOP2A | 1:1000 | Rabbit monoclonal | Abcam52934 |
| RRM2 | 1:1000 | Rabbit monoclonal | Abcam172476 |
| KIAA0101(PAF15) | 1:1000 | Rabbit monoclonal | Cell signaling technology(D8E2Y) |
| EXO1 | 1:1000 | Rabbit polyclonal | Abcam155553 |
| β-ACTIN | 1:1000 | Rabbit monoclonal | Cell signaling technology(13E5) |
